# Quantification of stress raisers in ARDS

**DOI:** 10.1186/cc12043

**Published:** 2013-03-19

**Authors:** M Cressoni, M Amini, P Cadringher, C Chiurazzi, D Febres, E Gallazzi, A Marino, M Brioni, F Menga, I Cigada, E Carlesso, D Chiumello, L Gattinoni

**Affiliations:** 1 Università degli Studi di Milano, Milan, Italy; 2 Fondazione IRCCS Ca' Granda-Ospedale Maggiore Policlinico, Milan, Italy

## Introduction

Ventilator-induced lung injury (VILI) is a well-known side effect of mechanical ventilation. The pressures and volumes needed to induce VILI in healthy animals are far greater than pressure and volumes applied in clinical practice [1]. A possible explanation may be the presence of local pressure multipliers (stress raisers).

## Methods

We retrospectively analyzed CT scans of 147 patients with ARDS and CT scans of 100 healthy subjects. A homogeneous lung would have the same gas/tissue ratio in all its regions. If a lung region expands less than the neighbour regions these will be more strained to vicariate the non/less expanding region. We measured the stress raisers by computing the ratio between the gas fraction of the region of interest and the neighbouring regions: if the inflation would be the same (homogeneity), the ratio will be equal to one; if the inflation of the surrounding regions would be greater than the region of interest (that is, more strained), the ratio between the two will be greater than one and was taken as a measure of stress raiser. We considered pathological stress raisers as the regions showing inflation ratio greater than the 95th percentile of the control group (1.61) and defined as the extent of the stress raisers the fraction of lung volume above this threshold.

## Results

The extent of stress raisers increased with the severity of ARDS (14 ± 5, 18 ± 8, 23 ± 1% of lung parenchyma in mild, moderate and severe ARDS, *P <*0.0001). The extent of stress raisers correlated with the dead space fraction (*r*^2 ^= 0.34, *P <*0.001), with the fraction of poorly aerated tissue (*r*^2 ^= 0.36, *P <*0.0001) and also has a negative correlation with the fraction of well inflated tissue (*r*^2 ^= 0.47, *P <*0.0001). The response to PEEP, passing from 5 to 45 cmH_2_O is minimal (average decrease of stress raiser extent 6 ± 5%) and inter-individual variability is great (in 11 patients, stress raisers increased passing from PEEP 5 to PEEP 45). Stress raisers turn out to be greater in nonsurvivor patients than in survivor patients (17 ± 7 vs. 20 ± 9% of lung volume, *P *= 0.03).

## Conclusion

Stress raisers correlate with the severity of disease; lungs with greater stress raisers are subjected to higher local pressures and probably those patients are more susceptible to VILI. The application of PEEP has little effect on stress raisers.
